# The adoption of an e-learning system using information systems success model: a case study of Jazan University

**DOI:** 10.7717/peerj-cs.723

**Published:** 2021-10-04

**Authors:** Bassam Al-shargabi, Omar Sabri, Shadi Aljawarneh

**Affiliations:** 1 Faculty of IT, Middle East University, Amman, Jordan; 2 Management Information System, Jazan University, Jazan, Saudi Arabia; 3 Faculty of IT, Jordan University of Science and Technology, Irbid, Jordan

**Keywords:** E-learning, IS success Model, DeLone and McLean Model, Stsudent’s Satisfaction, adoption of e-learning, higher education, online-learning, Saudi universities, E-readiness

## Abstract

**Background:**

The e-learning system has gained a phenomenal significance than ever before in the present COVID-19 crisis. The E-learning delivery mechanisms have evolved to enhanced levels facilitating the education delivery with greater penetration and access to mass student population worldwide. Nevertheless, there is still scope to conduct further research in order to innovate and improve higher quality delivery mechanism using the state-of-the-art information and communication technologies (ICT) available today. In the present pandemic crisis all the stakeholders in the higher education system, *i.e*., the governments, institutions, and the students expect seamless and efficient content delivery *via* e-learning platforms. This study proposes the adoption of the e-learning system by the integration of the model proposed by Delon and Mcclean “Information System Success Model” in Jazan University, Kingdom of Saudi Arabia (KSA) and further attempts to identify the factors affecting E-learning applications' success among the students.

**Methods:**

The data were gathered from 568 respondents. The Statistical Package for the Social Sciences version 26 (SPSS v.26.0) was used for the data analysis and one-way ANOVA is applied to test the hypothesis.

**Result:**

The overall results of this study allude to the fact that there is a significant relationship between Information system Success Model factors and the adoption of e-learning systems. The research results indicated that the information system success model has a strong associating cost-benefit value towards the adoption of e-learning systems across the Jazan University that may be further expanded to the other Saudi universities.

## Introduction

With the rapid growth of the Internet, prodigious information and communication technology (ICT) improvements have influenced practically all aspects of modern life. The information system (IS) has deeply penetrated and integrated into almost all the sectors ranging from organizations, industries, the education sector, and business activities in order to accomplish the desired objectives and to gain the associated benefits thereof (Ibrahim, 2018). The education sector is among such promising and lucrative sectors which are most impacted by technology adoption due to its enhanced capability of offering high-quality teaching. However, the e-learning environment is influenced by the degree of e-learning adoption (Lee & Lee, 2008; Al-Asmari & Rabb Khan, 2014). Presently, most universities and their managements worldwide have trusted in the IS and the Internet in their educational functions since the Internet has facilitated unbounded academic operations irrespective of geographical separations (Martins et al., 2019). Given that e-learning system is a blended combination of both students and instructors, the Internet usage by both has demonstrated that it can alter the traditional learning methods used with an interactive online system, yet most researches focus more on student perspectives (Wang, Solan & Ghods, 2010; Hassanzadeh, Kanaani & Elahi, 2012; Salam & Farooq, 2020; Martin, Modi & Feldman, 2021). The e-learning system (ELS) has now begun to play its role beyond teaching since it enables access to learning resources without any time or location limitations (Al-Fraihat et al., 2020). Teaching and learning systems have undergone phenomenal transformations over the past decade which is also established in the literature (Al-Asmari & Rabb Khan, 2014; Al-Harrasi, Al-Khanjari & Sarrab, 2015; Alhabeeb & Rowley, 2017; Martins et al., 2019). ELS in the education sector is being used globally for many years now. During the last two decades, e-learning is being used in communication course formats, audio-video tapes, videoconferencing, and TV broadcasts. Presently, the Internet is the optimal medium for e-learning; Internet-based distance education is considered to be the greatest common e-learning technological implementation of ICT (Martins et al., 2019). Also, the rapid growth in technology has gained traction due to the exponential growth usage of smart devices such as smartphones and hi-tech laptops at significant scales. Recent technologies and applications in smart devices have become the key elements of e-learning, communication, resource sharing, and management for both students and instructors. ELS such as Blackboard have completely redefined and transformed the traditional classes into web presentations. It provides direct links to conduct live classes, sessions, exams, upload and download files, discussions, and also enable students for asking questions and providing feedback (Liu, Huang & Lin, 2012; Alhomod & Shafi, 2013; Almaiah, Jalil & Man, 2016; Alksasbeh et al., 2019).

The Kingdom of Saudi Arabia (KSA) is one of the leading countries in the education sector that has implemented e-learning in light of the (COVID-19) pandemic. It has shifted the whole education processes over to e-learning systems. The Saudi government was being proactive in supporting and recommending the adoption of e-learning for both students and instructors through the workshops and training. Consequently, Jazan University was very proactive in implementing the government’s recommendations for all program courses delivery *via* the ELS. It has integrated e-learning into its educational processes for the majority of its courses. Intensive workshops and training sessions were conducted from the beginning for students and staff about the functioning of ELS (Blackboard). The results proved to have achieved the desired successful expectation which allowed the creation of dedicated departments and deanships to support and strengthen e-learning based educational systems (Martins et al., 2019; Salam & Farooq, 2020; Alhabeeb & Rowley, 2017; Duygu, Alkiş & Ozkan-Yildirim, 2018). Many Saudi universities are now integrating their learning processes and several applications systems such as management learning systems (MLS) and blackboard (Adeyinka & Mutula, 2010; Eom et al., 2012; Lin, 2013). Therefore, information and communication technology (ICT) has improved the collaboration between students and instructors, mutual interactions, management communications, and also educational performance as a whole. Additionally, many researchers have confirmed that ELS is a highly beneficial medium for distance education. ELS could be defined as “the combined use of modern information and communications technology (ICT) and computers to deliver instruction, information, and learning content” (Donovan et al., 2018). Alternatively, ELS is defined as a form of Internet technology based information system (IS) that delivers an unbounded, independent, and flexible training and learning opportunity to the learner (Duygu, Alkiş & Ozkan-Yildirim, 2018; Eom & Ashill, 2018). This technology-based system has rather simplified the learning processes (Al-Shargabi & Sabri, 2016).

The elements such as benchmarks, learning environment, learning outcomes, cost-benefit analysis, and IS models form the key constituents of an ELS. It is a combined scholarly suggestion for a necessity of a general model assessment and evaluation of e-learning programs eloquence (Liu, Huang & Lin, 2012; Alhomod & Shafi, 2013; Al-Harrasi, Al-Khanjari & Sarrab, 2015; Al-Shargabi & Sabri, 2016; Alhabeeb & Rowley, 2017; Yakubu & Dasuki, 2018; Alksasbeh et al., 2019). The scholars have proposed and evaluated models based on information system theory adapted from the information system success model (ISSM) and technology acceptance model (TAM), respectively (Liu, Huang & Lin, 2012; Alhomod & Shafi, 2013; Al-Harrasi, Al-Khanjari & Sarrab, 2015; Al-Shargabi & Sabri, 2016; Alhabeeb & Rowley, 2017; Yakubu & Dasuki, 2018; Alksasbeh et al., 2019). The outcome of their studies strongly urged for a necessity to implement an open-systems perspective based on general systems theory which operates on largely accepted concepts and principles with an arranged and interactive knowledge transfer (Wang, Solan & Ghods, 2010; Hassanzadeh, Kanaani & Elahi, 2012; Sabri, 2016; Opoku, Pobee & Owusu Okyireh, 2020; Martin, Modi & Feldman, 2021; Rouibah, Lowry & Almutairi, 2015; Marjanovic, Delić & Lalic, 2016).

The updated ISSM proposed by DeLone & McLean (1992) describes information systems success considering the factors of information quality, system, and services. These factors influenced the model usage and user satisfaction which eventually led to net benefits at reduced costs. The model has been used in the IS literature but sparingly in the ELS context. Hence, numerous studies endeavored to fill that gap by using IS variables of success model in the e-learning system context. This study is considered to be as one of the few studies that investigates university students’ e-learning adoption in Saudi Arabia’ by the implementation of ISSM (Al-Kofahi, Hassan & Mohamad, 2020; Ahmed & Seliaman, 2017). Therefore, it contributes to bridging the e-learning gap in the ELS studies which is already established in Saudi Arabia (Ahmed & Seliaman, 2017; Lin, 2013). As argued by DeLone & McLean (1992), the applications of IS are successful if businesses receive the net information systems benefits based on the usage of such systems and how satisfied the users are while using them. Therefore, this study is based on this model proposed by DeLone and McLean, to determine the success factors responsible for the ELS (Blackboard) acceptance at Jazan University where currently most of the universities in Saudi Arabic are shifting for online learning even after the disappearance of the COVID-19 pandemic. On the premise of the existing literature, background information, and the observed gap in the study, the research question for this study is: “What are the factors affecting the e-learning system to get the net cost-effective benefits at Jazan University?” Henceforth, the study was undertaken in order to corroborate the research question.

The rest of this paper is organized as follows: “Background” introduces the background of ISSM and ELS along with the most recent related work. “Materials and Methods” describes the data gathering, analysis, and hypothesis testing of the proposed model. The result and discussion of the proposed model for adoption of the E-learning system by the integration of the model proposed by Delon and McLean can be found in “Results and Discussion”. The conclusion was drawn and insight into future work are presented in the “Conclusion”.

## Background

A few studies with a limited scope have focused on the integration between the adoption of e-learning and the information system models, which touched upon the technical and management facets (Rouibah, Lowry & Almutairi, 2015; Marjanovic, Delić & Lalic, 2016; Martin, Modi & Feldman, 2021; Al-Kofahi, Hassan & Mohamad, 2020). This study attempts to provide a theoretical model from the information system and education fields. The theory used in this study includes the ISSM, which is associated with educational viewpoints and frequently deliberated in technology adoption studies. Before the introduction of this theory, a brief review of the current use of ELS and ISSM is provided as a background for the study.

### E-learning system (ELS)

Presently, the e-learning system is supposedly the most prevalent Internet based learning setting which aids in efficient time usage and boundary less learning (Sabri, 2016; Martin, Modi & Feldman, 2021). Nevertheless, these systems are successful subject the user acceptance and satisfaction. ELS users can access the system *via* Internet portals to reap the benefits of the information, lessons learned, knowledge, and skills. E-learners can access the courses either directly (live) or by the uploaded and posted content on the portal (accessed offline at a later time). These are later assessed by a variety of different methods on the knowledge obtained. This establishes the fact that ELS proves to be the best effective learning milieu. During the learning process, different system users have the flexibility of direct or indirect interactions with their peers (Alhomod & Shafi, 2013; Yakubu & Dasuki, 2018; Sabri, 2016; Opoku, Pobee & Owusu Okyireh, 2020; DeLone & McLean, 1992).

In the learning context, students and universities combined have several added advantages with the ELS service offerings. These advantages summarized as the following:

- Faster web access to information
- Enhanced upload and download contents
- Content standardization and accountability
- Availability
- Interactivity
- Confidence
- Higher user satisfaction
- Improved opportunities for career growth and flexible learning for students
- Increased innovation
- Superior operational efficiency and
- Cost savings

However, the advantages of ELS are dependent on users’ satisfaction, incessant system use, and intent to use. Therefore, it is highly imperative to comprehend users’ adoption to use ELS with reference to the predictive factors of behavioral intention theories (Selim, 2007; Sabri, 2016). These advantages aid universities to become more efficiently optimize their academic business procedures and operating costs. In order to determine the impacting factors towards developing the framework for this study, the analysis is undertaken for the previous works accomplished on models, case studies, and focus groups to investigate ELS miscellany (Al-Asmari & Rabb Khan, 2014; Alhabeeb & Rowley, 2017; Alhomod & Shafi, 2013; Alksasbeh et al., 2019; Yakubu & Dasuki, 2018). Owing to the ELS benefits and service offerings to universities and students, many universities have either increased their spending or allocated additional funds in order to upgrade the learning process systems. However, it is still vague to determine whether those factors had any influence on the success of ELS.

### Information system success model (ISSM)

**Figure 1:**
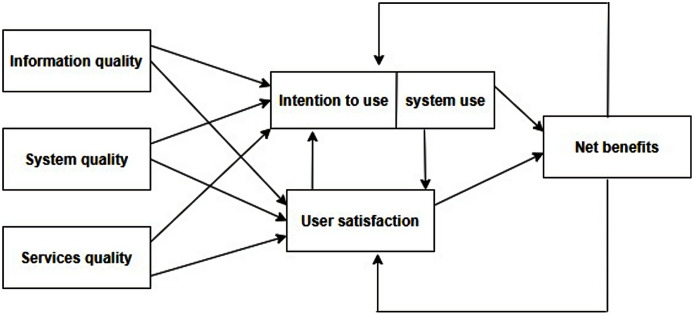
Updated DeLone and McLean information system success model (ISSM).

In 1992, DeLone & McLean (1992) had proposed an (ISSM) for measuring IS success in organizations to acquire the net benefit. They suggested that IS success is a multifaceted and symbiotic paradigm. Therefore, it is indispensable to study the interrelationships among those dimensions and control them. Subsequently, numerous scholars suggested some reforms to this model (Al-Shargabi & Sabri, 2016; Sabri, 2016; Altameem, 2013; Al-Shargabi & Sabri, 2015). Consequently, in 2003, DeLone & McLean (2016) incorporated some of the changes that scholars suggested and accordingly restructured their old model with the updated (ISSM) as illustrated in Fig. 1. They decided to augment the dimension of service quality, user satisfaction, intent to use, and net benefit thereof. The new model cited that service, system, information quality, system use, and user satisfaction are the critical success factors that lead to net benefits of using IS. The researchers argued if IS success evaluation is desired then, service, system, information, and quality is the impacting factor of its subsequent use. User satisfaction is the outcome of positive or negative benefits which will govern to promote the use of IS (DeLone & McLean, 2016).

Furthermore, this model illustrated in Fig. 1 is not limited to SME proprietor contentment only, yet it could be implemented and extended to placate the other users in industries, enterprises, and organizations by toting up other dynamics like hardware, software, network, security, policy, privacy, upper administration support, and structural traditional dynamics that influence IS evaluation (Kademeteme & Twinomurinzi, 2019; Adeyinka & Mutula, 2010; Li et al., 2012; Lin & Chen, 2012).

The updated ISSM and its success dimensions are listed as below:

- Information Quality: The level at which specific information obtained from the IS come to an agreement with the users’ anticipations and desires in terms of timeliness, accurateness, safety, significance, intuitiveness, and information consistency.
- System Quality: The level at which the IS system executes and functions aligned with the accord, convenience, simplicity of use, browsing, and learning, response time, and effectual consumption.

Service quality: The level at which the ICT center provides the system support which lays emphasis on the learning process and service accurateness and sustainability. While IS runs for steadiness, dependability, swift retrieval, speediness, and fortification of the communal networking system.

- System use: The function quality acuity for communal networking, proficient penetrating, information interchange, identity management, background cognizance, network cognizance, and contact management.
- User satisfaction: The degree of gratification provided to students regarding usefulness and efficacy by a comprehensive social networking quality system.
- Intention to use: The user’s learning alacrity to use and continue to use e-learning applications.
- Net benefits: It is the critical measure of triumph since it amplifies the negative and positive steadiness triumph of the system upon the users.

The IS recognizes the ISSM to be one of the most prominent models around. Further, its expediency has been quoted, verified, and established in several varied sectors including the academic institutions (Wang, Solan & Ghods, 2010; Martin, Modi & Feldman, 2021; Donovan et al., 2018; Al-Shargabi & Sabri, 2015; Hamidi & Jahanshaheefard, 2019). Notably, this is the most popular model ever established that has been in use on a frequent basis for testing the gratification levels of users, owners, and customers. Technology and human communication have a forecast divergence which is the subject of key disagreement between IS and universities (Kock, 2015; Al-Shargabi & Sabri, 2016; Yakubu & Dasuki, 2018). The universities’ key objective in establishing didactic guidance to both technology and management is based on e-learning acceptance by students’ affirmative attitude and willingness. Consequently, this study aims to offer an ISSM based evaluation model. Figure 1 above, illustrates the proposed model in this study for e-learning success evaluation from the student’s viewpoint. It describes and evolves measures of information, service, and system excellence and correlating impacts. There is an emergent requisite for e-learning adoption assessment based on previous studies and discourse. The objective of this study is focused on investigating student gratification with ELS’ adoption at Jazan University. The proposed model (as illustrated in Fig. 1) is deemed to be the most apposite model taking the developments in ELS adoption, Internet usage, and latest applications into account.

**Figure 2:**
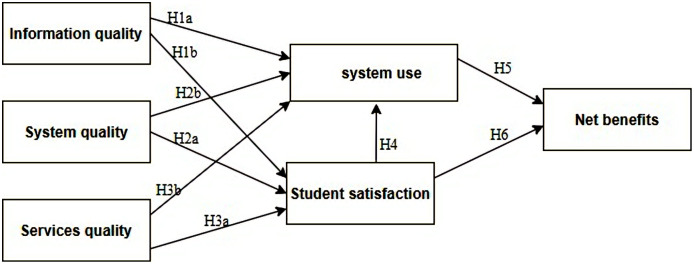
The theoretical framework for measuring of e-learning system students’ satisfaction.

This study has evolved six sets of ISSM based factors that contribute to e-learning satisfaction at Jazan University. Those factors (Fig. 2) are information quality, system quality, service quality, student satisfaction, system use, and net benefit to using ELS. According to the literature (Yakubu & Dasuki, 2018; Al Zoubib & Jali, 2014; Martins et al., 2019; Opoku, Pobee & Owusu Okyireh, 2020), it is believed that there is a scarcity in the literature case studies in Arab countries and further debate about the e-learning system with an Information system is a prerequisite. The implementation of the model should be bespoke according to the socio-economic, cultural, psychographic, geographic, and demographic conditions in the Arab countries in order to circumvent failures keeping in view the inferences of the literature (Alhomod & Shafi, 2013; Altameem, 2013; Al-Kofahi, Hassan & Mohamad, 2020; AlMulhem, 2020).

The primary research objective is to propose a research model which applies Information system adoption combined with literacy philosophies for further usage comprehension. To accomplish this objective, an empirical study was conducted on students at Jazan University, Saudi Arabia.

A systematic literature review of some articles related to e-learning systems adoption and information system success model (ISSM) is summarized as a Theoretical foundation as shown in Table 1 below. By utilizing the Scopus database. This study is based on 40 articles and conference papers from the years 2013 to 2021.

**Table 1:** The adoption of an E-learning system with an Information system success model perspective in the literature review.

Literature	The result	Year	Factors
(Saputra et al., 2020)	The study showed that the education quality and service quality in ISSM has positive effects on student’s satisfaction while using e-learning.	2020	Performance Expectancy; Effort System quality, information quality and service quality affects user satisfaction and actual usage
(Martins et al., 2019)	This model provides an elucidation to the incongruous literature outcomes regarding the overall user consummation quality *versus* usage as per actual. Finally, the study accomplished with the discoveries about the impact of technology disruption upon the behaviors of the online learners.	2019	System quality, information quality and service quality, user satisfaction, and actual usage
(Pérez-Pérez, Serrano-Bedia & García-Piqueres, 2020)	The findings of this study revealed that the quality of the information is the most proper indicator of students´ satisfaction, while satisfaction is the most applicable determining factor of perceived learning results.	2020	Course quality, system quality, information quality, and corresponding impacts.
(Alksasbeh et al., 2019)	An eminence attributes model for MSNs for higher education learning environment was suggested in this study.	2019	System quality, information quality, network quality, service quality, user satisfaction, and behavioral intention to use.
(AlMulhem, 2020)	The result of this study revealed that quality factors (course content quality, system quality, and service quality) have a positive and significant impact on students’ satisfaction with e-learning system quality.	2020	SD phase, MLD phase, LCD phase
(Yakubu & Dasuki, 2018)	The objective of this study was to test the acceptance factors for Canvas learning management system for the students of a private university in Nigeria.	2018	System quality, information quality, service quality, user satisfaction, behavioral intention, and actual usage
(Opoku, Pobee & Owusu Okyireh, 2020)	This was the first ever kind of study endeavored to analyze the acceptance of e-learning by the lecturers with the implementation of Information System Success and Technology Adoption Models in a University in Ghana.	2020	System quality, perceived usefulness, service quality, information quality, lecturers’ satisfaction, intention to use, and actual usage
(Sandjojo & Wahyuningrum, 2016)	The findings of this study showed that e-learning advantages can be effectively executed and clarified by its independent variables based on the information system model.	2016	System quality, information quality, service quality, user satisfaction, and behavioral intention
(Eom, 2013)	This study tested the predetermined IS success model in an e-learning context, which is a strictly involuntary use setting. The investigation of this study provided empirical proof to support an alternative DSS assessment model that can replace “system use” as the pivotal element of IS success model, with “perceived usefulness”	2013	System quality, perceived usefulness, service quality
(Effendy, Kurniawati & Priambada, 2021)	The results of this study showed that e-learning student satisfaction was impacted by service quality, information quality, and quality of education. Simultaneously, the intention to use is impacted by the quality of education. Likewise, user satisfaction and intention to use e-learning influence the real use of e-learning. This study is explored to aid leaders of higher education to more readily understand the adequacy of using e-learning by students.	2021	System quality, information quality, network quality, and service quality, user satisfaction, and behavioral intention to use.
(Ahmed & Seliaman, 2017)	Study results showed that the main indicators of students' continued intention to use E-learning systems are Perceived Benefits, Perceived Satisfaction, and Academic Motivation. The impact of each Task-technology fit, Performance expectancy, Knowledge Quality, and Information Quality on student's Perceived Satisfaction with utilizing E-learning systems was also additionally critical.	2017	System quality, perceived usefulness, service quality, information quality, lecturers’ satisfaction, intention to use, and actual usage
(Al Zoubib & Jali, 2014)	This study aimed to break down factors like compatibility, complexity, relative advantage, system quality, information quality, and service quality that drive adult workers in adopting an e-learning system in their learning process in Jordanian government universities. As a result, the Ministry of Higher Education in Jordan needs to have rules to help higher learning organizations to carry out e-learning effectively and proficiently by utilizing one of the successful IS models	2014	System quality, perceived usefulness, and service quality
			

## Materials & methods

The objective of this research is to examine the aspects accountable for the use of an ELS (Blackboard) application at Jazan University. Blackboard is the ELS being used in Jazan University that enables students to attend their classes online at the beginning of the semester, download course materials, engage in group discussions, submit assignments, and attend online quizzes and tests, and communicate with their instructors. A survey was developed and conducted in the study to generate quantitative descriptions of the respondents and test the relationship among the constructs (factors) used. The study’s respondents were Jazan University students because Students have shown and indicated that they were technologically adept and willing to employ any technological tool that may help them learn more effectively. This approach also have exploited and proved to be suitable as in Opoku, Pobee & Owusu Okyireh (2020). The data was gathered from 568 respondents and examined the hypotheses and the associated factors using SPSS (v26). One-way ANOVA is applied to test the hypothesis. A purposive sampling technique was used to select students who were using ELS. Data analyzed using the SPSS v 26 application. The study tested the hypothesis, checked for the reliability, and validity of the measurement model. A number of 27 items were designed, used, and analyzed for the study.

### Research model

This study has adopted the updated DeLone and McLean IS success model to examine the e-learning system’s net benefit from the students’ perspective to improve the understanding of e-learning adoption in Jazan University. This is a very popular model and easy to understand the technology adoption as discussed in the literature review. Hence, the main objectives of the research model are:

1. To identify the quality factors impacting e-learning system benefits in Jazan University.
2. To develop an empirically proposed model and provide support for the university to improve ELS and student benefits.

For this reason, it is necessary to copiously comprehend the relations between information systems success factors in ELS as shown in Fig. 2.

The proposed model illustrated in Fig. 2 examines the relationship between information quality, system quality, and service quality as antecedent variables that are expected to have a positive impact on user satisfaction and system use, which in turn affect the net benefit to use ELS by students who are using ELS. Therefore, the proposed model has six hypotheses to test:

H1a: Information quality will have a positive impact on system use.

H1b: Information quality will have a positive impact on student satisfaction

H2a: System quality will have a positive impact on student satisfaction.

H2b: System quality will have a positive impact on system use.

H3a: Service quality will have a positive influence on student satisfaction

H3b: Service quality will have a positive impact on system use

H4: Student satisfaction will have a positive impact on system use

H5: Student satisfaction will have a positive influence on the Net benefit of using ELS

H6: System use will have a positive impact on the Net benefit to use ELS

### Data collection

This study was conducted using both primary and secondary data. Firstly, the primary data based on e-learning and DeLone and McLean Information system success Model were collected. Literature reviews of 43 articles were accessed from several leading databases available at the Saudi Digital Library (SDL) such as Cambridge University Press, Emerald, EBSCOHost, Springer, Wiley Online, and Proquest. The cited articles were published within the range of 1992–2020. The secondary data were used to identify the independent variables discussed to affect the net benefit to ELS. The three independent variables are information quality (IQ), system quality (SQ), and service quality (Ser.Q) are the factors impacting system use and student satisfaction (St.S), which in turn impact the net benefit to using ELS (NBELS). To examine this, an online survey (Google form) contains a detailed questionnaire was created and sent out to students of Jazan university to answer the questionnaire which was used in the collection of primary data. The questionnaire was created using questions with validity and reliability of which have been solidly evidenced in papers in high-impact journals (Raman et al., 2018). Pre-testing of the questionnaire was conducted on a sample of 700 participants; a total number of 568 usable responses after excluding incomplete responses, resulting in a response rate of 81.147%. The survey questionnaire was divided into three-parts, the first part requested approval from the participants to confirm confidentiality, the second part focused on demographic information, and the third part contained statements concerning the conceptual framework of the study. A five-point Likert scale was used to measure the indicators (anchored at 1 = strongly disagree and 5 = strongly agree). Table 2 represented the demographic characteristic of the respondents where (*N* = 568).

**Table 2:** The demographic characteristics of the respondents.

Attributes	Category	Total		Male		Female	
		*N*	%	*N*	%	*N*	%
Age groups	Less than 20	111	19.54	81	14.26	30	5.28
	21–30	375	66.02	165	29.05	210	36.97
	31–40	45	7.92	27	4.75	18	3.17
	41–50	36	6.34	36	6.34	0	0.00
	51 and above	1	0.18	1	0.18	0	0.00
Education	First year	52	9.15	28	4.93	24	4.23
	Second year	72	12.68	39	6.87	58	10.21
	Third year	178	31.34	98	17.25	80	14.08
	Fourth year	154	27.11	90	15.85	64	11.27
	Fifth Year	58	10.21	35	6.16	23	4.05
	Sixth year	28	4.93	17	2.99	11	1.94

### Profile of the Respondents’

The demographic profile of the responses, split by gender frequency is shown in the Table 2 above.

#### Data analysis

Before conducting the data analysis, data must be screened to ensure the usability, validity, and reliability of the data before proceeding to the statistical analysis of such data. The data were collected on MS-EXCEL, where information was collect by using a structured questionnaire developed using Google forms for conducting an online Survey. 568 completed forms were selected and completed by respondents. In this paper, for analyzing the collected data, the Statistical Package for the Social Sciences version 26 (SPSS v.26.0) is exploited. The data analysis methods that we used in this paper are a descriptive, reliable, exploratory factor, correlation, and regression. These techniques are used for testing the proposed model based on students’ perceptions (Alksasbeh et al., 2019; Sabri, Hakim & Zaila, 2020).

#### Descriptive statistics of constructs

Descriptive Statistics is a prerequisite for a clear conceptualization of the constructs for the purpose of study. Table 3 below shows the descriptive statistics mean and standard deviation of each parameter within each construct (variables). All the questions which were measured on a five-point Likert Scale were transformed into Z-Scores with mean zero and standard deviation one. The results suggested that all the means were positive. The positive average Z-Score inferred that the respondent on the average (majority) have affirmatively responded.

**Table 3:** Summary of descriptive analysis.

Constructs	Items	Mean	Std. deviation
Information quality	IQ1	2.25	1.210
	IQ2	1.83	0.961
	IQ3	2.09	1.105
	IQ4	1.89	0.915
System quality	SQ1	2.40	1.284
	SQ2	1.87	0.986
	SQ3	2.37	1.275
	SQ4	2.00	1.198
Service quality	SerQ1	2.17	1.112
	SerQ2	2.42	1.123
	SerQ3	2.31	1.028
	SerQ4	2.48	1.079
Student satisfaction	StS1	2.15	1.310
	StS2	2.05	1.394
	StS3	2.08	1.377
	StS4	2.11	1.223
System use	Su1	2.08	1.377
	Su2	2.11	1.223
Net benefits to ELS	NBELS1	1.63	1.033
	NBELS2	1.79	1.067
	NBELS3	1.95	1.074
	NBELS4	2.00	1.316

### Model reliability and discriminant validity evaluation

The reflective model was utilized to ensure the reliability and validity of the construct measures and to provide support for the suitability of their inclusion in the path model. Following the recommendation in (Kock, 2015; Alksasbeh et al., 2019), measurement, reliability is assessed with Cronbach’s alpha coefficient, and Dillon–Goldstein rho coefficient (DG’s rho) to tests the internal consistency for items in the same construct. The accepted value of Cronbach’s alpha (α) must be the minimum threshold (0.70) as suggested by Nguyen (2020). As shown in Table 4, the Cronbach’s alpha value was more than 0.7. As (Chin, 1998) considers Dillon–Goldstein’s rho to be a better indicator than Cronbach’s alpha, Dillon-Goldstein’s rho values higher than 0.8 suggests unidimensional, which we have in the latent constructs. Besides, the higher value of Cronbach’s alpha coefficient and Dillon–Goldstein rho coefficient (DG’s rho) is a better indicator of a reflective construct which indicates satisfactory reliability for all five latent constructs. Here the bock is unidimensional, as the first eigenvalue is greater than one and the second is less than one. Again, Average Variance Extracted (AVE) of all constructs in the model exceeded 0.50, which is the recommended threshold (Fornell & Larcker, 1981). Further, Table 4 demonstrates an AVE of all the latent variables to be higher than its correlations. Thus the requirement of discriminant validity is also met.

**Table 4:** Summary of reliability analysis.

Items	Loadings		Reliability and convergent validity of the model					
	Cronbach alpha-item wise	Factor loadings-item wise	Cronbach’s alpha	Dillon-Goldstein’s rho	Eigen value-1	Eigen value-2	Average variance extracted (AVE)	R2
Information quality								
IQ1	0.97	0.84	0.84	0.86	2.72	0.60	0.68	0.00
IQ2	0.98	0.90						
IQ3	0.98	0.83						
IQ4	0.98	0.73						
System quality								
SQ1	0.98	0.87	0.91	0.91	3.14	0.33	0.79	0.70
SQ2	0.97	0.88						
SQ3	0.97	0.90						
SQ4	0.97	0.90						
Service quality								
SerQ1	0.98	0.91	0.94	0.94	3.40	0.30	0.62	0.35
SerQ2	0.98	0.92						
SerQ3	0.98	0.95						
SerQ4	0.98	0.91						
Student satisfaction								
Sts1	0.97	094	0.97	0.97	3.64	0.15	0.91	0.90
Sts2	0.97	0.96						
Sts3	0.97	0.96						
Sts4	0.97	0.95						
System use								
Su1	0.97	0.94	0.88	0.94	1.79	0.21	0.89	0.69
Su2	0.97	0.95						
Net benefit to use ELS								
NBLS1	0.98	0.84	0.91	0.91	3.12	0.43	0.78	0.75
NBLS2	0.97	0.88						
NBLS3	0.98	0.93						
NBLS4	0.97	0.89						

### Hypotheses testing: correlation and regression analysis

The proposed hypotheses are evaluated by using correlation and regression analysis. Correlation analysis examines the association among dependent and independent variables and hence determines the criteria to accept or reject the proposed hypotheses. In Table 5 below, the lower-triangular format depicts the correlation that establishes an association among the latent variables. The outcomes demonstrate the correlation to be greater than 0.50 (>0.50) which establishes a positive association among independent and dependent variables in the research model as shown in Table 5. Henceforth, it is obvious that outlier constructs are resilient and buttressed.

**Table 5:** Summary of correlation results.

Latent constructs	Information quality	System quality	Service quality	System use	Student satisfaction	Net benefit to use ELS
Latent constructs	1					
Latent constructs	0.758	1				
Latent constructs	0.589	0.725	1			
Latent constructs	0.839	0.886	0.639	1		
Latent constructs	0.815	0.782	0.584	0.906	1	
Latent constructs	0.733	0.788	0.620	0.851	0.836	1

The quality influencing factors on student satisfaction such as information, system, and service respectively are tested by using regression analysis which may impact the use of ELS. Hence, based on this the linear regression tested the initial regression model in Table 6. The outcomes demonstrated that information quality (H1, β = 0.451, *p* < 0.01), system quality (H2, β = 0.462, *p* < 0.01), service quality (H3, β = 0.242, *p* < 0.05) caused a significant impact on the student satisfaction and system use. Additionally, the dependent variable of student satisfaction R2 value is 0.872. This implies that there is a variance of 82.5% in the ELS student satisfaction which corroborates and explains information quality, system quality, and service quality impact factors within the proposed model.

**Table 6:** Summary of regression analysis.

Path co-efficient and model quality assessment									
Path	Hypotheses	*p*-Value	Path weight beta	Hypothesizes acceptance results	Decision	Direct effect	Indirect effect	Total effect	Relationship
iq -> seq	Higher the information quality, higher the service quality	<0.001**	0.59	YES	Supported	0.59	0.00	0.59	Moderate
iq -> sq	Higher the information quality, higher the system quality	<0.001**	0.52	YES	Supported	0.52	0.25	0.77	High
iq -> su	Higher the information quality, higher the system use	<0.001**	0.52	YES	Supported	0.52	0.27	0.79	High
iq -> ss	Higher the information quality, higher the student satisfaction	<0.001**	0.19	YES	Supported	0.19	0.65	0.84	High
seq -> sq	Higher the service quality, higher the system quality	<0.001**	0.42	YES	Supported	0.42	0.00	0.42	Moderate
seq -> su	Higher the service quality, higher the system use	<0.001**	0.06	YES	Supported	0.06	0.13	0.19	Low
sq -> su	Higher the system quality, higher the system use	<0.001**	0.31	YES	Supported	0.31	0.00	0.31	Moderate
sq -> ss	Higher the system quality, higher the student satisfaction	<0.001**	0.46	YES	Supported	0.46	0.11	0.57	Moderate
su -> ss	Higher the system use, higher the student satisfaction	<0.001**	0.38	YES	Supported	0.38	0.00	0.38	Moderate
su -> nf	Higher the system use, higher the net benefit to ELS	<0.001**	0.36	YES	Supported	0.36	0.20	0.56	Moderate
ss -> nf	Higher the student satisfaction, higher the net benefit to ELS	<0.001**	0.53	YES	Supported	0.53	0.00	0.53	Moderate

**Note:**

** *p*-Value obtained is too low such as (2.00E−69, 1.18E−47, etc.) to be reported.

Besides, the following hypotheses are also buttressed by the first-generation regression model:

H1a: Information quality will have a positive impact on system use.

H1b: Information quality will have a positive impact on student satisfaction

H2a: System quality will have a positive impact on student satisfaction.

H2b: System quality will have a positive impact on system use.

H3a: Service quality will have a positive influence on student satisfaction

H3b: Service quality will have a positive impact on system use

H4: Student satisfaction will have a positive impact on system use

H5: Student satisfaction will have a positive influence on the Net benefit of using ELS

H6: System use will have a positive impact on the Net benefit to use ELS

### Structural model path and testing of hypothesis

This research has investigated the causal relationship among the constructs. Here indirect effects denote mediation effects whereas the direct effect is the hypothesized relationship between two constructs. The sum of the direct effect and the indirect effect of a variable on another variable is called the total effect (Yakubu & Dasuki, 2018). The outcomes suggest that student satisfaction and system use gratification towards the implementation of e-learning system (ELS) are positively associated with information quality as information output in terms of accuracy, relevance, consistency, and completeness. Additionally, it is also evident and established that the system quality component which complies with the managerial decision-making characteristics of reliability, access, efficiency, and ease of use is also positively associated with student satisfaction and system use. Similarly, the service quality component which complies with the information technology adequacy measuring the client’s emotional evaluation of the expected satisfying service is certainly coupled with student satisfaction which is a performance measure of the system performance measurement. Table 6, illustrates the rest results of the specific hypothesis.

## Results and discussion

The primary contribution of this study is to be able to establish the notion that e-learning system implementation is impacted by ISSM which allows knowledge increment in further understanding of the ISSM model proposed by DeLone and McLean and how it impacts the e-learning system implementation in Saudi universities. The ISSM model is investigated in this study which leads to establishing the facts regarding the level of readiness of Saudi universities in order to implement ELS and further determining other contributing factors which are significant in positively impacting the successful ELS implementation in Saudi universities.

It is established from the results of this study that information quality has a positive effect on student satisfaction and system use. Videlicet, 75% strongly agree or partially agree on the awareness concept.

In system quality terms, the results suggest a positive impact on the readiness of Saudi universities towards ELS implementation. Principally, 82% of the participants either agree or partially agree to the impact of this factor on ELS implementation. Furthermore, the results corroborate nearly all the previous literature and allude to the advancement of commitment to change, chaos reduction, and resistance to change caused by the communication between the system and universities.

Additionally, results of this study have revealed that Saudi universities’ readiness to implement ELS is significantly impacted by service quality which is buttressed by 88% of respondents who either agree or strongly agree to the question. This is an indication to the point that older generations will encounter phenomenal challenges towards generating interest in adopting automation technologies. This also establishes that the higher the level of educational attainment in the society, the stronger the impacts of service quality on broadband adoption.

Besides, the odds of implementing ELS successfully are directly proportional to increased student satisfaction. Nevertheless, 18% of the respondents have negated based on the ELS privacy concerns and technology adoption and mellowness. Hence, it is of prime importance to address the issues of location-based applications and privacy violations. Besides, some respondents argued about the negative effects of ELS in terms of causing distractions and investing huge amounts of time adopting new applications and technology which is an arduous task and interferes with the traditional learning process.

Finally, the results revealed that Saudi universities’ readiness to implement ELS is also significantly impacted by system use which is buttressed by 82% of respondents who either agree. This is explained by the survey results based on the people’s awareness about the future of ELS in Saudi universities. This finding is in congruence and consistent with the researcher’s viewpoints that system use plays a critical role and determines the student’s behavior toward implementing ELS.

The results of this research study consistently buttress the literature (Martins et al., 2019; Salam & Farooq, 2020; Alhabeeb & Rowley, 2017; Duygu, Alkiş & Ozkan-Yildirim, 2018; Altameem, 2013; Maatuk et al., 2021; Aljawarneh et al., 2015). Unfortunately, there no researches regarding using ISSM for measuring adoption of ELS in Arab countries for comparing their outcomes with the obtained results in this study Furthermore, the outcomes of this endeavor can be utilized as a reference towards implementing ELS in other universities across Saudi Arabia or the Middle East and also by the other researchers and implementers in order to upgrade Arabic universities’ readiness levels in developing countries for new technologies adoption. Finally, this study can allow the top-level administrators to assist them in identifying the important factors affecting the success of implementation and introduction of a novel information system.

## Conclusions

The overall results of this study allude to the fact that there is a significant relationship between Information System Success Model factors and the adoption of e-learning systems. It is indicated that the study suggests that student satisfaction, system use, all quality components of information system and associated services are strongly leaned towards the implementation of e-learning system across the university. It is further revealed about Saudi universities’ readiness to implement ELS is also significantly impacted by system use. Therefore, the research results indicated that the information system success model has a strong associating cost-benefit value towards the adoption of e-learning systems across the Jazan University that may be further expanded to the other Saudi universities. The proposed recommendation in this study is to enhance system services to make students more agile in using the e-learning system.

## Supplemental Information

10.7717/peerj-cs.723/supp-1 Supplemental Information 1 Questionnaire.

10.7717/peerj-cs.723/supp-2 Supplemental Information 2 Dataset.

## Additional Information and Declarations

### Data Availability

The following information was supplied regarding data availability: Raw data is available as a Supplemental File.

## References

[ref-1] Adeyinka T, Mutula S (2010). A proposed model for evaluating the success of WebCT course content management system. Computers in Human Behavior.

[ref-2] Ahmed TM, Seliaman ME (2017). Investigating the adoption and impact of e-learning in KSA: Prince Sattam bin Abdulaziz university case study. Journal of Theoretical and Applied Information Technology.

[ref-48] Aljawarneh S, Alshargabi B, Hayajneh S, Imam A (2015). Integration of E-learning and cloud computing platform through software engineering. Recent Patents on Computer Science.

[ref-3] Al Zoubib AIS, Jali MZ (2014). An integrated success adoption model for examining E-learning among adult workers in Jordan.

[ref-4] Al-Asmari AM, Rabb Khan MS (2014). E-learning in Saudi Arabia: past, present and future. Near and Middle Eastern Journal of Research in Education.

[ref-5] Al-Fraihat D, Joy M, Masa’deh R, Sinclair J (2020). Evaluating E-learning systems success: an empirical study. Computers in Human Behavior.

[ref-6] Al-Harrasi H, Al-Khanjari Z, Sarrab M (2015). Proposing a new design approach for M-learning applications. International Journal of Software Engineering and its Applications.

[ref-7] Al-Kofahi MK, Hassan H, Mohamad R (2020). Information systems success model: a review of literature. International Journal of Innovation, Creativity and Change.

[ref-8] Al-Shargabi B, Sabri O (2015). An evaluation of MIS implementation success factors.

[ref-9] Al-Shargabi B, Sabri O (2016). A study of adopting cloud computing from enterprise perspective using DeLone and McLean IS success model. International Journal of Computer Science and Information Security.

[ref-10] Alhabeeb A, Rowley J (2017). Critical success factors for eLearning in Saudi Arabian universities. International Journal of Educational Management.

[ref-11] Alhomod S, Shafi MM (2013). Success factors of E-learning projects: a technical perspective. Turkish Online Journal of Educational Technology.

[ref-12] Alksasbeh M, Abuhelaleh M, Almaiah MA, Al-Jaafreh M, Karaka AA (2019). Towards a model of quality features for mobile social networks apps in learning environments: an extended information system success model. International Journal of Interactive Mobile Technologies.

[ref-13] Almaiah MA, Jalil MMA, Man M (2016). Empirical investigation to explore factors that achieve high quality of mobile learning system based on students’ perspectives. Engineering Science and Technology: An International Journal.

[ref-14] AlMulhem A (2020). Investigating the effects of quality factors and organizational factors on university students’ satisfaction of e-learning system quality. Cogent Education.

[ref-15] Altameem A (2013). What drives successful e-learning? An empirical investigation of the key technical issues in Saudi Arabian universities. Journal of Theoretical and Applied Information Technology.

[ref-16] Chin WW (1998). Issues and opinion on structural equation modeling. MIS Quarterly: Management Information Systems.

[ref-17] DeLone WH, McLean ER (1992). Information systems success: the quest for the dependent variable. Information Systems Research.

[ref-18] DeLone WH, McLean ER (2016). Information systems success measurement. Foundations and Trends® in Information Systems.

[ref-19] Donovan E, Indira GR, Adya M, Wang W (2018). A cloud update of the DeLone and McLean model of information systems success. Journal of Information Technology Management.

[ref-20] Duygu DC, Alkiş N, Ozkan-Yildirim S (2018). A structural model for students’ adoption of learning management systems: an empirical investigation in the higher education context. Educational Technology and Society.

[ref-50] Effendy F, Kurniawati OD, Priambada G (2021). Factor affecting E-Learning user acceptance: a case study of AULA. Journal of Physics: Conference Series.

[ref-53] Eom S (2013). Testing the seddon model of information system success in an e-learning context: Implications for evaluating DSS. Lecture Notes in Business Information Processing.

[ref-21] Eom SB, Ashill NJ (2018). A system’s view of E-learning success model. Decision Sciences Journal of Innovative Education.

[ref-22] Eom S, Ashill NJ, Arbaugh JB, Stapleton JL (2012). The role of information technology in e-learning systems success. Human Systems Management.

[ref-23] Fornell C, Larcker DF (1981). Evaluating structural equation models with unobservable variables and measurement error. Journal of Marketing Research.

[ref-24] Hamidi H, Jahanshaheefard M (2019). Essential factors for the application of education information system using mobile learning: A case study of students of the university of technology. Telematics and Informatics.

[ref-25] Hassanzadeh A, Kanaani F, Elahi S (2012). A model for measuring e-learning systems success in universities. Expert Systems with Applications.

[ref-26] Ibrahim A (2018). Factors affecting the adoption of ICT by administrators in the university for development studies tamale: empirical evidence from the UTAUT model. International Journal of Sustainability Management and Information Technologies.

[ref-27] Kademeteme E, Twinomurinzi H (2019). A structural equation model for the evaluation of the switching costs of information communication technology in SMEs. Electronic Journal of Information Systems Evaluation.

[ref-28] Kock N (2015). Common method bias in PLS-SEM: a full collinearity assessment approach. International Journal of e-Collaboration.

[ref-29] Lee JK, Lee WK (2008). The relationship of e-learner’s self-regulatory efficacy and perception of e-Learning environmental quality. Computers in Human Behavior.

[ref-30] Li Y, Duan Y, Fu Z, Alford P (2012). An empirical study on behavioural intention to reuse e-learning systems in rural China. British Journal of Educational Technology.

[ref-31] Lin TC (2013). Combining the TAM and IS success models to validate e-learning system satisfaction and continuance intention.

[ref-32] Lin TC, Chen CJ (2012). Validating the satisfaction and continuance intention of e-learning systems: combining tam and is success models. International Journal of Distance Education Technologies.

[ref-33] Liu YC, Huang YA, Lin C (2012). Organizational factors’ effects on the success of e-learning systems and organizational benefits: an empirical study in Taiwan. International Review of Research in Open and Distance Learning.

[ref-47] Maatuk AM, Elberkawi EK, Aljawarneh S, Rashaideh H, Alharbi H (2021). The COVID-19 pandemic and E-learning: challenges and opportunities from the perspective of students and instructors. Journal of Computing in Higher Education.

[ref-34] Marjanovic U, Delić M, Lalic B (2016). Developing a model to assess the success of e-learning systems: evidence from a manufacturing company in transitional economy. Information Systems and e-Business Management.

[ref-35] Martin HD, Modi SS, Feldman SS (2021). Barriers and facilitators to PDMP IS Success in the US: a systematic review. Drug and Alcohol Dependence.

[ref-36] Martins J, Branco F, Gonçalves R, Au-Yong-Oliveira M, Oliveira T, Naranjo-Zolotov M, Cruz-Jesus F (2019). Assessing the success behind the use of education management information systems in higher education. Telematics and Informatics.

[ref-37] Nguyen OT (2020). Factors affecting the intention to use digital banking in Vietnam. Journal of Asian Finance, Economics and Business.

[ref-38] Opoku D, Pobee F, Owusu Okyireh R (2020). Determinants of E-learning system adoption among ghanaian university lecturers: an application of information system success and technology acceptance models. American Journal of Social Sciences and Humanities.

[ref-49] Pérez-Pérez M, Serrano-Bedia AM, García-Piqueres G (2020). An analysis of factors affecting students’ perceptions of learning outcomes with Moodle. Journal of Further and Higher Education.

[ref-39] Raman S, Patwa N, Niranjan I, Ranjan U, Moorthy K, Mehta A (2018). Impact of big data on supply chain management. International Journal of Logistics Research and Applications.

[ref-40] Rouibah K, Lowry PB, Almutairi L (2015). Dimensions of business-to-consumer (B2C) systems success in Kuwait: testing a modified DeLone and McLean IS success model in an e-commerce context. Journal of Global Information Management.

[ref-41] Sabri O (2016). Applying the updated DeLone and McLean is success model for enterprise cloud computing readiness. International Journal of Cloud Applications and Computing.

[ref-42] Sabri O, Hakim T, Zaila B (2020). The role of hofstede dimensions on the readiness of IoT implementation case study: Saudi universities. Journal of Theoretical and Applied Information Technology.

[ref-43] Salam M, Farooq MS (2020). Does sociability quality of web-based collaborative learning information system influence students’ satisfaction and system usage?. International Journal of Educational Technology in Higher Education.

[ref-52] Sandjojo N, Wahyuningrum T (2016). Measuring e-learning systems success: Implementing D and M is success model.

[ref-51] Saputra R, Rayana T, Adhy S, Bahtiar N, Timu ME (2020). Success factor affecting M-Learning implementation: perspective of students.

[ref-44] Selim HM (2007). Critical success factors for e-learning acceptance: confirmatory factor models. Computers and Education.

[ref-45] Wang J, Solan D, Ghods A (2010). Distance learning success—a perspective from socio-technical systems theory. Behaviour and Information Technology.

[ref-46] Yakubu MN, Dasuki SI (2018). Assessing eLearning systems success in Nigeria: an application of the DeLone and McLean information systems success model. Journal of Information Technology Education: Research.

