# Supplementary material for: The adoption of an e-learning system using information systems success model: a case study of Jazan University
Source: PeerJ Comput Sci. 2021 Oct 4;7:e723. doi: 10.7717/peerj-cs.723 (PMC8507483; doi:10.7717/peerj-cs.723)
Supplement: Supplemental Information 1 [file peerj-cs-07-723-s001.pdf]

Dear Sir/Madam,

Greetings from Omar Sabri, Bassam Al-Shargabi, and Shadi Aljawarneh, we are working on a research titled "***The Adoption of E-learning System Using Information Systems Success Model: A Case Study of Jazan University***"

The aim of this study to measures the success of Jazan University in applying the e-learning system Blackboard, by applying the updated Information system success model on the Jazan university system.

All information in this study will be used for academic research purpose only. Your response will be kept in strict confidence.

Thank you in advance for your involvement in this Survey. We hope that the results of this study will provide useful insights to future implementations and improvements of the e-learning system in (Jazan Universities).

If you have any questions or concerns about this questionnaire or the study, please do not hesitate to contact us: (+966) 594299411 or email me at osabri@jazanu.edu.sa

Thank you very much in advance for taking the time to answer the questions in the survey. We really appreciate you!

Researchers:

Omar Sabri

Bassam Al-Shargabi

Shadi Aljawarneh

1. **General questions**

Please kindly answer the following questions:

Gender

- Male
- Female

Age

- Less than 20
- Less than 30
- Less than 40
- Less than 50
- Less Than 60

Educational Year

- First year
- Second year
- Third year
- Forth year
- Fifth year
- Six year
- Other

2. Research questionnaire about user perceptions of e-learning at Jazan University

Please indicate your level of agreement with the following statements:

1 = Strongly Agree, 2 = Agree, 3 = Neutral, 4 = Disagree, 5 Strongly Disagree

Questions related to the dimensions of the study

	Sentences	1	2	3	4	5
**A-**	**Quality of information provided by E-learning System**					
	**The quality of information measures the quality of the lecture resources and lessons that have been obtained through the Blackboard e-learning system in terms of: synchronization,**					

	**availability of information, ease of understanding, appropriateness of information to the nature of the course, completeness of information according to the course, and safety.**					
1	In general, we can say that the information I obtain through the Blackboard e-learning system is complete and accurate					
2	In general, we can say that the information I obtain through the Blackboard e-learning system is related to my study topics in the specialization					
3	In general, we can say that the information I obtain through the Blackboard e-learning system is easy to understand					
4	In general, we can say that the information I get from Blackboard e-learning is safe					
	**B- Quality of E-learning System**					
	**System quality measures the required characteristics of an e-learning system, these characteristics include ease of use, responsiveness, adaptability, and reliability.**					
5	We can say that the e-learning system using Blackboard is always available					
6	In general, we can say that the e-learning system using Blackboard is reliable					
7	We can say that the response time with the blackboard e-learning system is very good					
8	We can say that the e-learning system using Blackboard is easy to adapt					
	**C- Quality of Service provided by E-learning System**					
	**It measures the quality of service provided by the technical support unit by the developers of the e-learning system such as the IT department, and these characteristics include: knowledge, empathy, responsiveness, and effectiveness.**					
9	The technical support staff of the Blackboard e-learning system have the required resources to ensure that the system is allowed to be used at all times					
10	Blackboard e-learning technical support staff provides a rapid response service to my requests					
11	Blackboard e-learning technical support staff have the necessary knowledge for my requests when I need them					
12	Blackboard e-learning technical support staff shows sympathy towards me while responding to my requests					
	**D- Student satisfaction with the Service provided by E-learning System**					
	**User satisfaction measures the extent to which the individual perceives the initial expectations of using the e-learning system. These characteristics include: reliability, importance, usefulness and effectiveness. It has been shown that users' satisfaction with e-learning systems affects behavioral intentions.**					
13	I am satisfied with all the services and functions provided by the Blackboard e-learning system that is currently used					
14	The e-learning system made the teaching process easier for me					
15	In general, I am satisfied with the use of the Blackboard e-learning system					
16	I am satisfied with the support I get while using the Blackboard e-learning system					
	**E- Intention to use E-learning system**					

	**Behavioral intent measures the likelihood that an individual will engage in a particular behavior in the context of e-learning, there is a relationship between behavioral intent and actual use of e-learning systems.**					
17	I intend to use the Blackboard e-learning system in the coming semesters					
18	I intend to encourage other students to use the e-learning system					
19	I expect that I will use the e-learning system in the coming classes					
20	It is recommended to use e-learning systems for teaching					
**F-**	**Net Benefit of using E-learning system**					
	**The ultimate benefit of the e-learning system's benefits measures the user's behavior of the e-learning system**					
21	I expect that the use of the e-learning system will reduce expenses					
22	I expect that the use of the e-learning system in the coming years will be expanded in all universities					
23	I expect that the use of the e-learning system will reduce the time of the scientific research process during my studies					
24	I expect that using the e-learning system will save me the time needed to study and work					
